# FUT8-AS1 Inhibits the Malignancy of Melanoma Through Promoting miR-145-5p Biogenesis and Suppressing NRAS/MAPK Signaling

**DOI:** 10.3389/fonc.2020.586085

**Published:** 2021-05-19

**Authors:** Xiang-jun Chen, Sha Liu, Dong-mei Han, De-zhi Han, Wei-jing Sun, Xiao-chun Zhao, Jun-qing Liang, Li Yu

**Affiliations:** ^1^Department of Burns and Plastic Surgery, The 969^th^ Hospital of PLA, Hohhot, China; ^2^The Affiliated People's Hospital of Inner Mongolia Medical University, Inner Mongolia Autonomous Region Cancer Hospital, Hohhot, China; ^3^Department of Intensive Care Unit, The 969^th^ Hospital of PLA, Hohhot, China

**Keywords:** FUT8-AS1, melanoma, malignancy, NF90, microRNA biogenesis, MAPK signaling

## Abstract

Melanoma is the major lethal skin malignancy. However, the critical molecular drivers governing melanoma progression and prognosis are still not clear. By analyzing The Cancer Genome Atlas (TCGA) data, we identified FUT8-AS1 as a prognosis-related long non-coding RNA (lncRNA) in melanoma. We further confirmed that FUT8-AS1 is downregulated in melanoma. Reduced expression of FUT8-AS1 is correlated with aggressive clinical factors and inferior overall survival. Using *in vitro* functional assays, our findings demonstrated that ectopic expression of FUT8-AS1 represses melanoma cell proliferation, migration, and invasion. FUT8-AS1 silencing promotes melanoma cell proliferation, migration, and invasion. Furthermore, *in vivo* functional assays demonstrated that FUT8-AS1 represses melanoma growth and metastasis. Mechanistically, FUT8-AS1 was found to bind NF90, repress the interaction between NF90 and primary miR-145 (pri-miR-145), relieve the repressive roles of NF90 on mature miR-145-5p biogenesis, and thus promote miR-145-5p biogenesis and upregulate mature miR-145-5p level. The expression of FUT8-AS1 is positively correlated with miR-145-5p in melanoma tissues. *Via* upregulating miR-145-5p, FUT8-AS1 reduces the expression of NRAS, a target of miR-145-5. FUT8-AS1 further represses MAPK signaling *via* downregulating NRAS. Functional rescue assays demonstrated that inhibition of miR-145-5p reverses the tumor suppressive roles of FUT8-AS1 in melanoma. The oncogenic roles of FUT8-AS1 silencing are also blocked by MAPK signaling inhibitor MEK162. In conclusion, these findings demonstrate that FUT8-AS1 exerts tumor suppressive roles in melanoma *via* regulating NF90/miR-145-5p/NRAS/MAPK signaling axis. Targeting FUT8-AS1 and its downstream molecular signaling axis represent promising therapeutic strategies for melanoma.

## Introduction

Despite the total cancer incidence rate is declined since 1991, the incidence rate of skin melanoma is still increasing for both male and female since 1975 (1). Therefore, skin melanoma has been the fifth most common cancer in male, accounting for 7% of all site cancers according to 2020 cancer statistics of USA (1). In female, skin melanoma is the sixth most common cancer and accounts for 4% of all site cancers according to 2020 cancer statistics of USA (1). Although recent approval of molecule targeted therapy and immunotherapy for melanoma has greatly extended the survival of melanoma patients, most late melanomas particular for those with metastases are still incurable (2, 3). Thus, uncovering the critical molecular drivers responsible for the progression and poor survival of melanomas would enable more effective therapy for melanoma.

Recently, many high-throughput RNA sequencings and systems biology approaches have revealed the critical roles of non-coding RNA (ncRNA) in tumorigenesis and progressions (4–6). Among these ncRNAs, long non-coding RNA (lncRNA) and microRNA (miRNA) present significantly aberrant expression, clinical reverences, and important roles in various malignancies (7–10). lncRNA is a class of long ncRNAs with more than 200 nucleotides (nt) in length, and while miRNA is a class of short ncRNAs with 19–25 nt in length (11–14). Increasing knowledge of lncRNA and miRNA has revealed that aberrant expression of lncRNA and miRNA would be potential prognostic and diagnostic biomarkers for malignancies (15–17). Furthermore, many lncRNAs and miRNAs also show oncogenic or tumor suppressive roles, which are involved in almost every aspect of cancers, including the initiation, proliferation, apoptosis, cell cycle, senescence, stemness, migration, invasion, drug-resistance, and so on (18–20).

In melanoma, the knowledge of miRNAs is relatively abundant. Many oncogenic or tumor suppressive miRNAs have been identified, such as the oncogenic miR-410-3p, miR-21-5p, miR-125b-5p, and miR-378a-5p (21–24) and the tumor suppressive miR-107, miR-140-5p, miR-204-5p, and miR-128-3p (25–28). In our previous report, we also found that miR-145-5p is downregulated in melanoma and suppresses melanoma cell proliferation, migration, and invasion *in vitro*, and melanoma tumor growth *in vivo* (29). The major mechanism of action of miRNAs is to bind AGO2 and form RNA-induced silencing complex (RISC), which further binds target mRNAs and induces target mRNAs degradation and/or translation inhibition (30). In our previous report, we also identified NRAS as a direct target of miR-145-5p, which further modulates MAPK signaling (29).

In comparison, the knowledge of lncRNAs in melanoma is relative less (31). The contributions of lncRNAs to melanoma initiation and progression are only starting to be studied (32). Several cancer-related lncRNAs in melanoma were revealed, such as SLNCR1, OVAAL, EMICERI, THOR (33–36). In our previous reports, we also identified three melanoma-related lncRNAs, including PVT1, ILF3-AS1, and MHENCR (37–39). The mechanisms of action of lncRNAs are complex and various. Some lncRNAs directly bind proteins and modulates the expression and/or functions of interacted proteins, such as OVAAL, THOR, and ILF3-AS1 (34, 36, 38). Some lncRNAs directly bind miRNAs and repress the roles of interacted miRNAs, such as MHENCR (39). Some lncRNAs may also directly modulate neighboring protein-coding genes, such as EMICERI (35). Despite several lncRNAs have been elucidated in melanoma, the contributions of most other lncRNAs to melanoma are still unclear. As transcriptomic sequencings have identified more than 58,000 lncRNAs in human (40), we could not preclude the contributions of other lncRNAs to melanoma.

To further identify the lncRNAs involved in melanoma, we analyzed The Cancer Genome Atlas (TCGA) Skin Cutaneous Melanoma (SKCM) dataset, and searched the genes correlated with outcome of melanoma patients. *FUT8-AS1* was identified as one of the most significantly correlated genes with melanomas’ prognosis. In this study, we further investigated the expression and clinical reverence of FUT8-AS1 in melanoma. *In vitro* and *in vivo* gain- and loss-of-function assays were performed to elucidate the biological roles of FUT8-AS1 in melanoma. Furthermore, the mechanism of action of FUT8-AS1 was explored and we identified a relative novel mechanism of action of FUT8-AS1, which is the promotion of miR-145-5p biogenesis.

## Materials and Methods

### Human Tissue Samples

A total of 68 malignant melanoma tissues and 36 age and gender-matched skin tissues with melanocytic nevi were acquired from patients who had undergone surgical resection at the 969^th^ Hospital of PLA (Hohhot, Inner Mongolia, China). All tissue samples were diagnosed by histopathological examination. The Review Board of the 969^th^ Hospital of PLA approved this study. Written informed consents were obtained from all patients.

### Cell Culture and Treatment

Human melanoma cell lines CHL-1 and SK-MEL-2 were acquired from Cell Resource Center, Chinese Academy of Sciences. CHL-1 and SK-MEL-2 cells were cultured in DMEM and MEM medium respectively supplemented with 10% fetal bovine serum (Gibco, Thermo Fisher Scientific). Where indicated, cells were treated with 1 µM MEK162 (Selleck) for indicated time.

### RNA Isolation and Real-Time PCR

Total RNA was isolated from indicated tissues and cells by Trizol reagent (Invitrogen, Thermo Fisher Scientific) in accordance with the manufacturer’s manual. The first strand cDNA was generated using the RNA and the PrimeScript™ II 1st Strand cDNA Synthesis Kit (Takara, Dalian, China). Real-time PCR was performed using TB Green^®^ Premix Ex Taq™ II (Takara) on StepOnePlus Real-Time PCR System (Thermo Fisher Scientific) with the primers 5’-GGCTCCTTGCTACTTTTAGGG-3’ (forward) and 5’-TGGGGGGGGTCTTTCTCTTC-3’ (reverse) for FUT8-AS1, 5’-GAAATACGCCAGTACCGAATG-3’ (forward) and 5’-TTCTCCTCCAGGGAAGTCAG-3’ (reverse) for NRAS, 5’-GTCGGAGTCAACGGATTTG-3’ (forward) and 5’-TGGGTGGAATCATATTGGAA-3’ (reverse) for GAPDH. GAPDH was used as endogenous control for the quantification of FUT8-AS1 and NRAS expression. For the quantification of miRNAs and pri-miRNAs expression, real-time PCR was carried out using the TaqMan™ Advanced miRNA Assay (Thermo Fisher Scientific) and TaqMan™ Pri-miRNA Assay (Thermo Fisher Scientific) respectively on StepOnePlus Real-Time PCR System in accordance with the manufacturer’s manuals.

### Construction of Stable Cell Lines

To construct melanoma cells stably overexpressing FUT8-AS1, FUT8-AS1 overexpressing lentivirus (LV11/CMV/Neo) were acquired from GenePharma (Shanghai, China) and infected into CHL-1 and SK-MEL-2 cells. Next, the cells were treated with neomycin for 4 weeks to select CHL-1 and SK-MEL-2 cells overexpressing FUT8-AS1. Two pairs of cDNA oligonucleotides inhibiting FUT8-AS1 expression were designed and generated by GenePharma. After annealing, double-strand oligonucleotides were inserted into the shRNA lentiviral vector pLV6/EF-1a/Puro to produce shRNA lentivirus inhibiting FUT8-AS1 expression. A scrambled non-targeting shRNA was used as negative control (NC). Next, CHL-1 and SK-MEL-2 cells were infected with the shRNA lentivirus. The cells were treated with puromycin for 4 weeks to select FUT8-AS1 silenced cells. The shRNA sequences were as follows: 5’-GATCCGCCCTACTTTATCTTGTAAGATTCAAGAGATCTTACAAGATAAAGTAGGGCTTTTTTG-3’ (forward) and 5’-AATTCAAAAAAGCCCTACTTTATCTTGTAAGATCTCTTGAATCTTACAAGATAAAGTAGGGCG-3’ (reverse) for LV-shRNA-1, 5’-GATCCGCGGAAGTTTATTTAGTACGGTTCAAGAGACCGTACTAAATAAACTTCCGCTTTTTTG-3’ (forward) and 5’-AATTCAAAAAAGCGGAAGTTTATTTAGTACGGTCTCTTGAACCGTACTAAATAAACTTCCGCG-3’ (reverse) for LV-shRNA-2, 5’-GATCCGTTCTCCGAACGTGTCACGTTTCAAGAGAACGTGACACGTTCGGAGAACTTTTTTG-3’ (forward) and 5’-AATTCAAAAAAGTTCTCCGAACGTGTCACGTTCTCTTGAAACGTGACACGTTCGGAGAACG-3’ (reverse) for LV-shNC. To construct melanoma cells overexpressing FUT8-AS1 and concurrently inhibiting miR-145-5p, FUT8-AS1 overexpressed CHL-1 cells were infected with miR-145-5p inhibiting lentivirus (Genechem Co. Ltd., Shanghai, China) and treated with neomycin and puromycin for 4 weeks to select FUT8-AS1 overexpressed and concurrently miR-145-5p inhibited cells.

### Cell Viability, Proliferation, Migration, and Invasion Assays

Glo cell viability assay was performed to measure cell viability as previously described (38). Briefly, 3,000 indicated melanoma cells per well were plated into 96-well plates. At the indicated time, the luminescence values were detected by the Cell Titer-Glo Luminescent Cell Viability Assay (Promega) to record cell viability. Ethynyl deoxyuridine (EdU) incorporation assay was performed using the EdU Kit (Roche) to measure cell proliferation as previously described (38). Transwell migration assay was performed to measure cell migration as previously described (38). Transwell invasion assay was performed to measure cell invasion as previously described (38).

### Xenografts in Nude Mice

Athymic BALB/c nude mice were purchased from Chinese Academy of Sciences and maintained in pathogen-free condition. The use of animals was approved by the Review Board of the 969^th^ Hospital of PLA (Hohhot, Inner Mongolia, China). A total of 3.0 × 10^6^ indicated melanoma cells were subcutaneously injected into the flanks of nude mice. Subcutaneous xenograft volumes were detected using caliper every 7 days and calculated following the formula V = 0.5 × LW2 (L, tumor length; W, tumor width). At the 28^th^ day after injection, subcutaneous xenografts were resected and weighed. Subcutaneous xenografts were further used to carry out immunohistochemistry (IHC) staining with the primary antibody against Ki67 (ab15580, 1 µg/ml, Abcam). Subcutaneous xenografts were also used to perform TdT-mediated dUTP Nick-End Labeling (TUNEL) staining using the One Step TUNEL Apoptosis Assay Kit (Beyotime, Shanghai, China). To evaluate melanoma liver metastasis *in vivo*, 3.0 × 10^6^ indicated melanoma cells were intrasplenically injected into nude mice to construct liver metastasis model. At the 28^th^ day after injection, the mice were sacrificed and the livers were resected. Hematoxylin-eosin (H&E) staining was performed using the livers. To evaluate melanoma lung metastasis *in vivo*, 3.0 × 10^6^ indicated melanoma cells were injected into tail vein of nude mice to construct lung metastasis model. At the 28^th^ day after injection, the mice were sacrificed and the lungs were resected. H&E staining was performed using the lungs.

### RNA Pull-Down Assay

FUT8-AS1 full-length sequences were PCR-amplified with the primers 5’-GGAATTCTCGCTGCGCCGGTGGAGA-3’ (forward) and 5’-GCTCTAGATTTCAGTTGGAAGGAGGTAGG-3’ (reverse). The PCR products were cloned into the EcoR I and Xba I sites of pSPT19 vector (Roche) to construct pSPT19-FUT8-AS1. NF90 binding sites mutated pSPT19-FUT8-AS1 (pSPT19-FUT8-AS1-mut) was constructed using the Fast Mutagenesis System (TransGen Biotech, Beijing, China) with the primers 5’-CTTTATCTTGTAAGAGGACAATCCACATTCAC-3’ (forward) and 5’-TTGTCCTCTTACAAGATAAAGTAGGGCTTCG-3’ (reverse). Wild type and NF90 binding sites mutated FUT8-AS1 was *in vitro* transcribed and biotinylated from pSPT19-FUT8-AS1 and pSPT19-FUT8-AS1-mut, respectively, using the Biotin RNA Labeling Mix (Roche) and Sp6 RNA polymerase (Roche). After purification, 3 µg of wild type or NF90 binding sites mutated FUT8-AS1 were incubated with 1 mg of whole-cell lysates from CHL-1 cells at 25°C for 1 h. The streptavidin agarose beads (Thermo Fisher Scientific) were used to enrich biotinylated RNA and interacted proteins. The proteins present in the pull-down material were detected using western blot.

### Western Blot

Western blot was conducted as previously described (38) with the primary antibodies: for NF90, ab131004, 1:2,000, Abcam; for EZH2, #07-689, 1:2,000, Millipore; for NRAS, ab154291, 1:1,000, Abcam; for GAPDH, ab8245, 1:5,000, Abcam; for phospho-MEK1/2, #9154, 1:1,000, Cell Signaling Technology; for MEK1/2, #8727, 1:1,000, Cell Signaling Technology; for phospho-ERK1/2, #4370, 1:2,000, Cell Signaling Technology; for ERK1/2, #4695, 1:1,000, Cell Signaling Technology.

### RNA Immunoprecipitation (RIP)

RIP assays were performed in indicated melanoma cells using the Magna RIP RNA-Binding Protein Immunoprecipitation Kit (Millipore) and an antibody against NF90 (5 µg per reaction; ab131004, Abcam) in accordance with the manufacturer’s manual.

### Statistical Analysis

GraphPad Prism v6.0 was employed to perform all statistical analyses. For comparisons, log-rank test, Mann-Whitney test, two-tailed unpaired t test, one-way ANOVA followed by Dunnett’s multiple comparisons test, Spearman correlation analysis, or one-way ANOVA followed by Tukey’s multiple comparisons test was conducted as indicated in figure legends. *P* < 0.05 was considered as statistically significant.

## Results

### Reduced Expression of FUT8-AS1 Is Correlated With Inferior Prognosis of Melanoma

**Figure 1:**
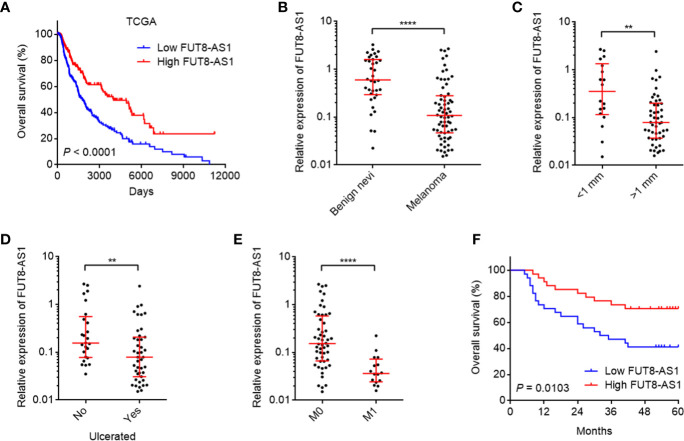
The expression and clinical relevance of FUT8-AS1 in melanoma. **(A)** Kaplan-Meier survival curves of skin cutaneous melanoma (SKCM) from TCGA dataset stratified by FUT8-AS1 expression (low 50% [n = 229] *versus* high 50% [n = 229]). *P* < 0.0001 by log-rank test. **(B)** FUT8-AS1 expression in 36 benign nevi and 68 melanoma tissues was measured by real-time PCR. **(C)** FUT8-AS1 expression in 18 melanoma tissues with thickness <1 mm and 50 melanoma tissues with thickness >1 mm. **(D)** FUT8-AS1 expression in 25 melanoma tissues without ulceration and 43 melanoma tissues with ulceration. **(E)** FUT8-AS1 expression in 52 melanoma tissues without distant metastasis and 16 melanoma tissues with distant metastasis. For **(B–E)**, data are presented as median with interquartile range. **P < 0.01, ****P < 0.0001 by Mann-Whitney test. **(F)** Kaplan-Meier survival curves of 68 melanomas stratified by FUT8-AS1 expression (low 50% [n = 34] *versus* high 50% [n = 34]). *P* = 0.0103 by log-rank test.

To search the genes correlated with prognosis of melanoma, we analyzed The Cancer Genome Atlas (TCGA) Skin Cutaneous Melanoma (SKCM) dataset to retrieve the differentially expressed genes between different vital statuses (*P*-value < 0.01, fold change ≥2, **Supplementary Table 1**). Among these differentially expressed genes, we noted FUT8-AS1, which has a relative more significant *P*-value and bigger fold change. Further analysis of the TCGA-SKCM dataset revealed that reduced expression of FUT8-AS1 indicated inferior overall survival (**Figure 1A**). Two *in silico* tools, namely the Coding Potential Assessment Tool (CPAT) (http://lilab.research.bcm.edu/cpat/index.php) and the Coding Potential Calculator (CPC) (http://cpc2.cbi.pku.edu.cn/) were employed to calculate the coding potential of FUT8-AS1. CPC and CPAT scores of FUT8-AS1 were equally low as well-known lncRNA HOTAIR (**Supplementary Figures 1A, B**), which indicated the noncoding nature of FUT8-AS1. Next, we measured FUT8-AS1 expression in our own cohort including 36 benign nevi and 68 melanomas using real-time PCR. The results indicated that FUT8-AS1 was downregulated in melanoma tissues compared with benign nevi (**Figure 1B**). Correlation analyses between FUT8-AS1 expression and clinicopathological characters indicated that reduced expression of FUT8-AS1 was correlated with thickness, ulceration, and metastasis (**Figures 1C–E**). Moreover, Kaplan-Meier survival analysis indicated that reduced expression of FUT8-AS1 was correlated with inferior overall survival among these 68 melanomas (**Figure 1F**). Thus, these findings suggest that FUT8-AS1 is downregulated in melanoma and reduced expression of FUT8-AS1 is correlated with aggressive clinical factors and inferior overall survival.

### FUT8-AS1 Inhibits Melanoma Cell Proliferation, Migration, and Invasion *In Vitro*

**Figure 2:**
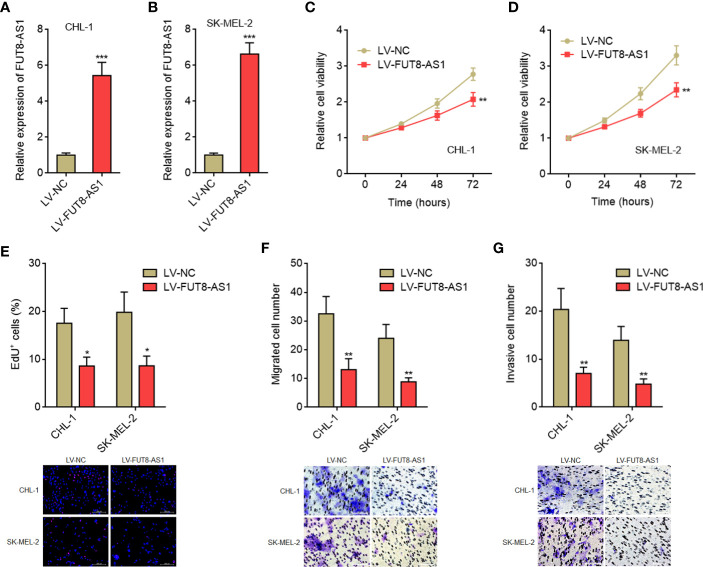
FUT8-AS1 inhibits melanoma cell proliferation, migration, and invasion. **(A)** FUT8-AS1 expression in CHL-1 cells overexpressing FUT8-AS1or control was measured by real-time PCR. **(B)** FUT8-AS1 expression in SK-MEL-2 cells overexpressing FUT8-AS1 or control was measured by real-time PCR. **(C)** Cell viabilities of CHL-1 cells overexpressing FUT8-AS1 or control were determined by the Glo cell viability assay. **(D)** Cell viabilities of SK-MEL-2 cells overexpressing FUT8-AS1 or control were determined by the Glo cell viability assay. **(E)** Cell proliferation of CHL-1 and SK-MEL-2 cells overexpressing FUT8-AS1 or control were determined by the EdU incorporation assay. The red color indicates EdU-positive nuclei. Scale bars, 200 µm. **(F)** Cell migration of CHL-1 and SK-MEL-2 cells overexpressing FUT8-AS1 or control were determined by the transwell migration assay. Scale bars, 100 µm. **(G)** Cell invasion of CHL-1 and SK-MEL-2 cells overexpressing FUT8-AS1 or control were determined by the transwell invasion assay. Scale bars, 100 µm. Data are presented as mean ± SD. *P < 0.05, **P < 0.01, ***P < 0.001 by two-tailed unpaired t test.

Due to the significant correlation between FUT8-AS1 expression and clinical characteristics of melanoma, we next investigated the potential roles of FUT8-AS1 in melanoma. We constructed CHL-1 and SK-MEL-2 cells stably overexpressing FUT8-AS1 *via* FUT8-AS1 overexpression lentivirus mediated transfection. The overexpression efficiencies were confirmed by real-time PCR (**Figures 2A, B**). Glo cell viability assays indicated that both CHL-1 and SK-MEL-2 cells overexpressing FUT8-AS1 had reduced cell viabilities compared with their control cells, respectively (**Figures 2C, D**). EdU incorporation assays further indicated that both CHL-1 and SK-MEL-2 cells overexpressing FUT8-AS1 had slower cell proliferation rates compared with their control cells, respectively (**Figure 2E**). Transwell migration assays indicated that both CHL-1 and SK-MEL-2 cells overexpressing FUT8-AS1 had less migrated cell numbers compared with their control cells, respectively (**Figure 2F**). Transwell invasion assays indicated that both CHL-1 and SK-MEL-2 cells overexpressing FUT8-AS1 had less invasive cell numbers compared with their control cells, respectively (**Figure 2G**). Thus, these findings suggest that FUT8-AS1 inhibits melanoma cell proliferation, migration, and invasion *in vitro*.

**Figure 3:**
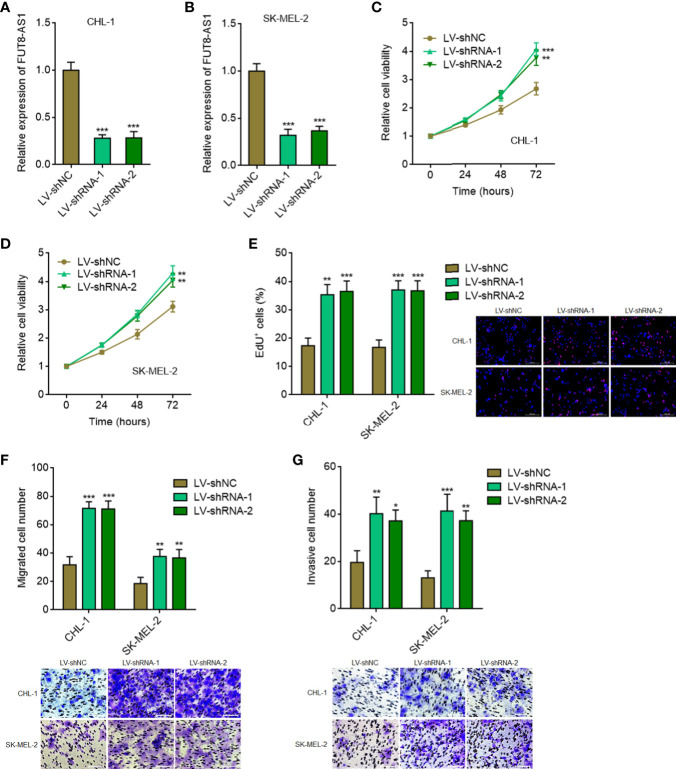
FUT8-AS1 silencing promotes melanoma cell proliferation, migration, and invasion. **(A)** FUT8-AS1 expression in CHL-1 cells silencing FUT8-AS1 or control was measured by real-time PCR. **(B)** FUT8-AS1 expression in SK-MEL-2 cells silencing FUT8-AS1 or control was measured by real-time PCR. **(C)** Cell viabilities of CHL-1 cells silencing FUT8-AS1 or control were determined by the Glo cell viability assay. **(D)** Cell viabilities of SK-MEL-2 cells silencing FUT8-AS1 or control were determined by the Glo cell viability assay. **(E)** Cell proliferation of CHL-1 and SK-MEL-2 cells silencing FUT8-AS1 or control were determined by the EdU incorporation assay. The red color indicates EdU-positive nuclei. Scale bars, 200 µm. **(F)** Cell migration of CHL-1 and SK-MEL-2 cells silencing FUT8-AS1 or control were determined by the transwell migration assay. Scale bars, 100 µm. **(G)** Cell invasion of CHL-1 and SK-MEL-2 cells silencing FUT8-AS1 or control were determined by the transwell invasion assay. Scale bars, 100 µm. Data are presented as mean ± SD. *P < 0.05, **P < 0.01, ***P < 0.001 by one-way ANOVA followed by Dunnett’s multiple comparisons test.

To further confirm the tumor suppressive roles of FUT8-AS1 in melanoma, we constructed FUT8-AS1 stably silenced CHL-1 and SK-MEL-2 cells *via* two independent FUT8-AS1 specific shRNAs lentivirus mediated transfection (**Figures 3A, B**). Glo cell viability assays indicated that FUT8-AS1 silenced CHL-1 and SK-MEL-2 cells both had increased cell viabilities compared with their control cells, respectively (**Figures 3C, D**). EdU incorporation assays indicated that FUT8-AS1 silenced CHL-1 and SK-MEL-2 cells both had quicker cell proliferation rates compared with their control cells, respectively (**Figure 3E**). Transwell migration assays indicated that FUT8-AS1 silenced CHL-1 and SK-MEL-2 cells both had more migrated cell numbers compared with their control cells, respectively (**Figure 3F**). Transwell invasion assays indicated that FUT8-AS1 silenced CHL-1 and SK-MEL-2 cells both had more invasive cell numbers compared with their control cells, respectively (**Figure 3G**). Collectively, these findings suggest that FUT8-AS1 silencing promotes melanoma cell proliferation, migration, and invasion *in vitro*, further supporting the tumor suppressive roles of FUT8-A1 in melanoma.

### FUT8-AS1 Inhibits Melanoma Growth and Metastasis *In Vivo*

**Figure 4:**
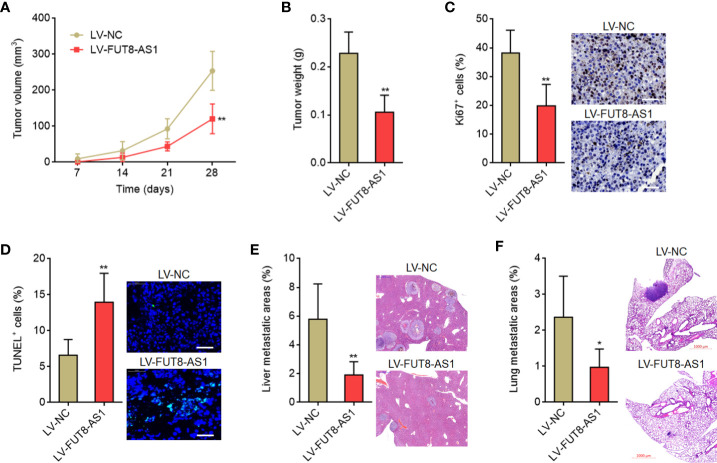
FUT8-AS1 inhibits melanoma growth and metastasis *in vivo*. **(A, B)** CHL-1 cells overexpressing FUT8-AS1 or control were subcutaneously inoculated into nude mice. Tumor volumes were measured every 7 days **(A)**. The tumors were excised and weighed at the 28^th^ day after inoculation **(B)**. **(C)** The tumors formed by CHL-1 cells overexpressing FUT8-AS1 or control were used to perform Ki67 IHC staining. Scale bars, 50 µm. **(D)** The tumors formed by CHL-1 cells overexpressing FUT8-AS1 or control were used to perform TUNEL staining. Scale bars, 50 µm. **(E)** CHL-1 cells overexpressing FUT8-AS1 or control were inoculated into spleen of nude mice to construct liver metastasis model. The livers were excised and used to perform H&E staining. Scale bars, 500 µm. **(F)** CHL-1 cells overexpressing FUT8-AS1 or control were inoculated into tail vein of nude mice to construct lung metastasis model. The lungs were excised and used to perform H&E staining. Scale bars, 1,000 µm. Data are presented as mean ± SD. n = 6 mice in each group. *P < 0.05, **P < 0.01 by Mann-Whitney test.

To investigate the roles of FUT8-AS1 in melanoma growth *in vivo*, FUT8-AS1 stably overexpressed and control CHL-1 cells were subcutaneously inoculated into nude mice. Tumor volumes were measured every 7 days and the tumors were excised and weighed at the 28^th^ day after inoculation. The results indicated that the tumors formed by CHL-1 cells overexpressing FUT8-AS1 had a slower growth rate and formed a smaller tumor compared with the tumors formed by control cells (**Figures 4A, B**). Proliferation marker Ki67 IHC staining indicated that the tumors formed by CHL-1 cells overexpressing FUT8-AS1 had less Ki67 positive cells compared with the tumors formed by control cells (**Figure 4C**). TUNEL staining indicated that the tumors formed by CHL-1 cells overexpressing FUT8-AS1 had more apoptotic cells compared with the tumors formed by control cells (**Figure 4D**). To investigate the roles of FUT8-AS1 in melanoma metastasis *in vivo*, CHL-1 cells overexpressing FUT8-AS1 or control were intrasplenically injected to construct liver metastasis model. The results indicated that CHL-1 cells overexpressing FUT8-AS1 formed less liver metastases compared with control CHL-1 cells (**Figure 4E**). Furthermore, CHL-1 cells overexpressing FUT8-AS1 or control were injected into the tail veins of nude mice to construct lung metastasis model. The results indicated that CHL-1 cells overexpressing FUT8-AS1 formed less lung metastases compared with control CHL-1 cells (**Figure 4F**). Thus, these findings suggest that FUT8-AS1 inhibits melanoma growth and metastasis *in vivo*, further supporting the tumor suppressive roles of FUT8-AS1 in melanoma.

### FUT8-AS1 Enhance miR-145-5p Biogenesis *via* Binding NF90

**Figure 5:**
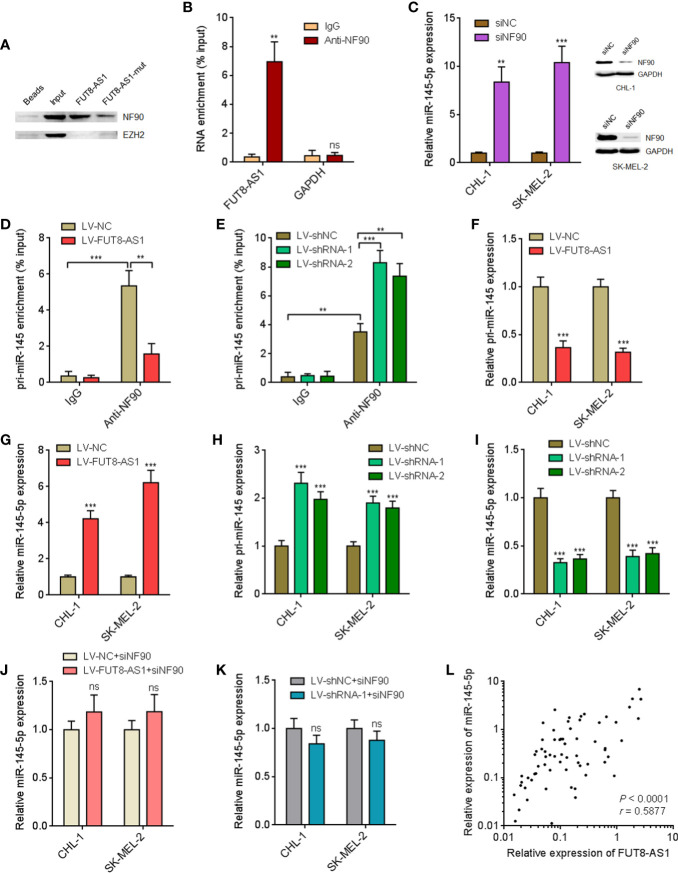
FUT8-AS1 upregulates miR-145-5p *via* binding NF90. **(A)** RNA pull-down assays using *in vitro* transcribed wild-type or NF90 binding site mutant FUT8-AS1. The enriched proteins were measured by western blot. **(B)** RIP assays were performed in CHL-1 cells using NF90 specific antibody or non-specific IgG. The enriched RNA was detected by real-time PCR. **(C)** After transfection of NF90 specific siRNAs pool or non-targeting negative control (NC) siRNAs into CHL-1 and SK-MEL-2 cells, miR-145-5p expression levels and NF90 protein expression levels were measured by real-time PCR and western blot, respectively. **(D)** RIP assays were performed in SK-MEL-2 cells overexpressing FUT8-AS1 or control using NF90 specific antibody or non-specific IgG. The enriched RNA was detected by real-time PCR. **(E)** RIP assays were performed in CHL-1 cells silencing FUT8-AS1 or control using NF90 specific antibody or non-specific IgG. The enriched RNA was detected by real-time PCR. **(F)** pri-miR-145 expression levels in CHL-1 and SK-MEL-2 cells overexpressing FUT8-AS1 or control were measured by real-time PCR. **(G)** Mature miR-145-5p expression levels in CHL-1 and SK-MEL-2 cells overexpressing FUT8-AS1 or control were measured by real-time PCR. **(H)** pri-miR-145 expression levels in CHL-1 and SK-MEL-2 cells silencing FUT8-AS1 or control were measured by real-time PCR. **(I)** Mature miR-145-5p expression levels in CHL-1 and SK-MEL-2 cells silencing FUT8-AS1 or control were measured by real-time PCR. **(J)** After transfection of NF90 specific siRNAs pool into CHL-1 and SK-MEL-2 cells overexpressing FUT8-AS1 or control, mature miR-145-5p expression levels were measured by real-time PCR. **(K)** After transfection of NF90 specific siRNAs pool into CHL-1 and SK-MEL-2 cells silencing FUT8-AS1 or control, mature miR-145-5p expression levels were measured by real-time PCR. **(L)** miR-145-5p expression levels in the same 68 melanoma tissues used in **Figure 1** were measured by real-time PCR. The correlation between miR-145-5p and FUT8-AS1 expression levels in these 68 melanoma tissues was calculated by Spearman correlation analysis. r = 0.5877, *P* < 0.0001. For **(B–K)**, data are presented as mean ± SD. **P < 0.01, ***P < 0.001, ns, not significant, by two-tailed unpaired t test [**B**, **C**, **D**, **E** (the comparison between IgG and Anti-NF90 for LV-shNC), **F**, **G**, **J**, **K**] or one-way ANOVA followed by Dunnett’s multiple comparisons test [**E** (the comparison between LV-shRNA-1, LV-shRNA-2, and LV-shNC for Anti-NF90), **H**, **I**].

Many lncRNAs were shown to exert their biological roles *via* interacting with proteins (41). To identify whether FUT8-AS1 could also interact with proteins, we used the online *in silico* tool RNA-Protein Interaction Prediction (RPISeq) (http://pridb.gdcb.iastate.edu/RPISeq/) to predict the potential interaction between FUT8-AS1 and proteins. Notably, we predicted a potential interaction between FUT8-AS1 and NF90 with an interaction probability of 0.95. FUT8-AS1 contains a conserved NF90 binding sequence (5’-CUGUU-3’, 452-456nt of FUT8-AS1, **Supplementary Figure 2**), supporting the potential interaction between FUT8-AS1 and NF90. NF90 has been revealed to repress miR-145-5p biogenesis *via* binding pri-miR-145 (42). Our previous report had revealed the tumor suppressive role of miR-145-5p in melanoma (29). Therefore, we next investigated the potential effects of FUT8-AS1 on NF90/miR-145-5p. RNA pull-down assays using *in vitro* transcribed biotin-labeled FUT8-AS1 showed the specific enrichment of NF90, but not EZH2 (**Figure 5A**). In addition, the enrichment of NF90 by FUT8-AS1 was significantly reduced by the mutation of NF90 binding sequence in FUT8-AS1 (**Figure 5A**). RIP assays with NF90 specific antibody showed the enrichment of FUT8-AS1, but not GAPDH mRNA (**Figure 5B**), which further supports the interaction between FUT8-AS1 and NF90. To investigate whether NF90 regulates miR-145-5p biogenesis in melanoma and whether FUT8-AS1 modulates the effects of NF90 on miR-145-5p, we first detected the effects of NF90 on miR-145-5p. Silencing of NF90 significantly upregulated miR-145-5p expression in both CHL-1 and SK-MEL-2 cells (**Figure 5C**). RIP assays with NF90 specific antibody revealed the significant enrichment of pri-miR-145 in both CHL-1 and SK-MEL-2 cells (**Figures 5D, E**), supporting the binding of NF90 to pri-miR-145 in melanoma. Furthermore, overexpression of FUT8-AS1 significantly decreased the binding between pri-miR-145 and NF90 in SK-MEL-2 cells (**Figure 5D**). Conversely, silencing of FUT8-AS1 increased the binding between pri-miR-145 and NF90 in CHL-1 cells (**Figure 5E**). These data suggested that FUT8-AS1 reduced the binding of NF90 to pri-miR-145. Next, we measured pri-miR-145 and mature miR-145-5p expression levels in FUT8-AS1 overexpressed CHL-1 and SK-MEL-2 cells. The results indicated that pri-miR-145 was reduced in FUT8-AS1 overexpressed CHL-1 and SK-MEL-2 cells compared with their control cells (**Figure 5F**), and while mature miR-145-5p was significantly increased in FUT8-AS1 overexpressed CHL-1 and SK-MEL-2 cells (**Figure 5G**). Furthermore, pri-miR-145 and mature miR-145-5p expression levels in FUT8-AS1 silenced CHL-1 and SK-MEL-2 cells were measured. The results indicated that pri-miR-145 was increased in FUT8-AS1 silenced CHL-1 and SK-MEL-2 cells compared with their control cells (**Figure 5H**), and while mature miR-145-5p was significantly reduced in FUT8-AS1 silenced CHL-1 and SK-MEL-2 cells (**Figure 5I**). To elucidate whether the regulation of miR-145-5p by FUT8-AS1 is dependent on NF90, we depleted NF90 in FUT8-AS1 overexpressed and silenced CHL-1 and SK-MEL-2 cells, and then measured mature miR-145-5p expression levels. The results revealed that depletion of NF90 abolished the effects of FUT8-AS1 overexpression and silencing on miR-145-5p (**Figures 5J, K**). Collectively, these findings suggest that FUT8-AS1 interacted with NF90, relieved the repressive roles of NF90 on miR-145-5p biogenesis, and therefore downregulated pri-miR-145 and upregulated mature miR-145-5p levels in melanoma. The positive correlation between FUT8-AS1 and miR-145-5p expression levels was also found in melanoma tissues (**Figure 5L**).

### FUT8-AS1 Represses NRAS/MAPK Signaling

**Figure 6:**
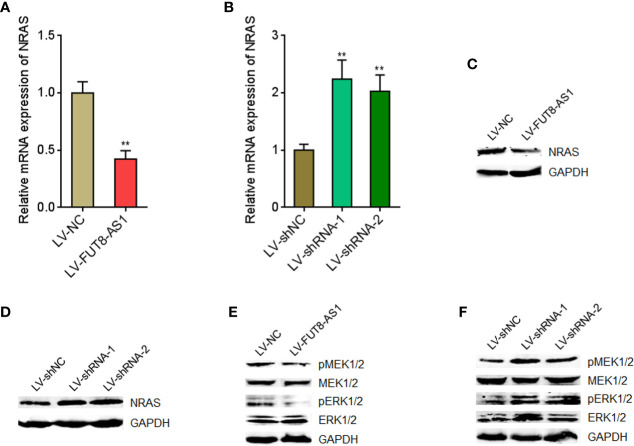
FUT8-AS1 represses NRAS/MAPK signaling. **(A)** NRAS mRNA expression levels in SK-MEL-2 cells overexpressing FUT8-AS1 or control were measured by real-time PCR. **(B)** NRAS mRNA expression levels in CHL-1 cells silencing FUT8-AS1 or control were measured by real-time PCR. **(C)** NRAS protein levels in SK-MEL-2 cells overexpressing FUT8-AS1 or control were measured by western blot. **(D)** NRAS protein levels in CHL-1 cells silencing FUT8-AS1 or control were measured by western blot. **(E)** Phosphorylation levels of MEK1/2 and ERK1/2 in SK-MEL-2 cells overexpressing FUT8-AS1 or control were measured by western blot. **(F)** Phosphorylation levels of MEK1/2 and ERK1/2 in CHL-1 cells silencing FUT8-AS1 or control were measured by western blot. Data are presented as mean ± SD. **P < 0.01 by two-tailed unpaired t test **(A)** or one-way ANOVA followed by Dunnett’s multiple comparisons test **(B)**.

In our previous report, we have found that miR-145-5p activated MAPK signaling *via* directly targeting NRAS (29). Therefore, we further investigated the effects of FUT8-AS1 on NRAS/MAPK signaling. Our findings revealed that NRAS mRNA levels were decreased in FUT8-AS1 overexpressed cells and increased in FUT8-AS1 silenced cells (**Figures 6A, B**). Consistently, NRAS protein levels were decreased in FUT8-AS1 overexpressed cells and increased in FUT8-AS1 silenced cells (**Figures 6C, D**). Next, the effects of FUT8-AS1 on MAPK signaling were investigated. As shown in **Figures 6E, F**, the phosphorylation levels of MEK1/2 and ERK1/2 were decreased in FUT8-AS1 overexpressed cells and increased in FUT8-AS1 silenced cells (**Figures 6E, F**). Thus, these data suggest that FUT8-AS1 represses NRAS/MAPK signaling in melanoma.

### The Tumor Suppressive Roles of FUT8-AS1 Are Dependent on the Regulation of miR-145-5p/NRAS/MAPK Signaling Axis

**Figure 7:**
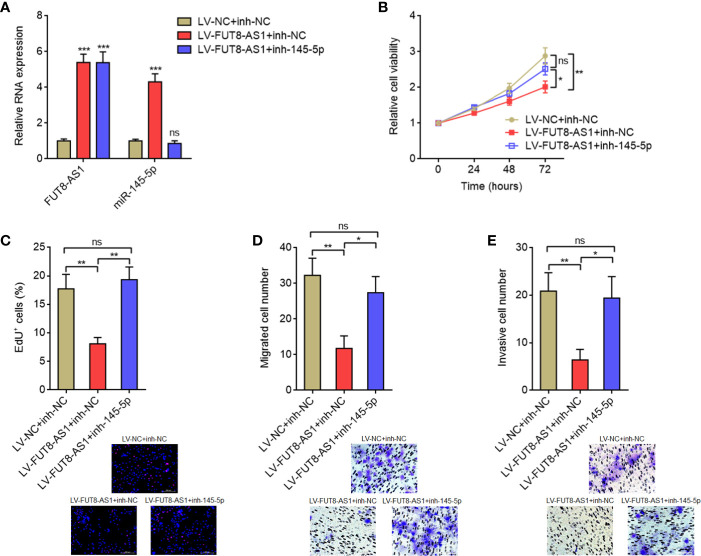
miR-145-5p inhibition reversed the tumor suppressive roles of FUT8-AS1 in melanoma. **(A)** FUT8-AS1 and miR-145-5p expression levels in CHL-1 cells overexpressing FUT8-AS1 and concurrently silencing miR-145-5p were measured by real-time PCR. **(B)** Cell viabilities of CHL-1 cells overexpressing FUT8-AS1 and concurrently silencing miR-145-5p were determined by the Glo cell viability assay. **(C)** Cell proliferation of CHL-1 cells overexpressing FUT8-AS1 and concurrently silencing miR-145-5p were determined by the EdU incorporation assay. The red color indicates EdU-positive nuclei. Scale bars, 200 µm. **(D)** Cell migration of CHL-1 cells overexpressing FUT8-AS1 and concurrently silencing miR-145-5p were determined by the transwell migration assay. Scale bars, 100 µm. **(E)** Cell invasion of CHL-1 cells overexpressing FUT8-AS1 and concurrently silencing miR-145-5p were determined by the transwell invasion assay. Scale bars, 100 µm. Data are presented as mean ± SD. *P < 0.05, **P < 0.01, ***P < 0.001; ns, not significant, by one-way ANOVA followed by Tukey’s multiple comparisons test.

To elucidate whether the tumor suppressive roles of FUT8-AS1 in melanoma were dependent on the regulation of miR-145-5p/NRAS/MAPK signaling axis, we inhibited miR-145-5p in FUT8-AS1 overexpressed CHL-1 cells (**Figure 7A**). Glo cell viability assays indicated that the reduced cell viabilities caused by FUT8-AS1 overexpression were reversed by miR-145-5p inhibition (**Figure 7B**). EdU incorporation assays indicated that the slower cell proliferation rates caused by FUT8-AS1 overexpression were reversed by miR-145-5p inhibition (**Figure 7C**). Transwell migration assays indicated that the reduced migrated cell number caused by FUT8-AS1 overexpression were reversed by miR-145-5p inhibition (**Figure 7D**). Transwell invasion assays indicated that the reduced invasive cell number caused by FUT8-AS1 overexpression was reversed by miR-145-5p inhibition (**Figure 7E**). Collectively, these findings showed that the suppressive roles of FUT8-AS1 in melanoma cell proliferation, migration, and invasion were reversed by miR-145-5p inhibition.

**Figure 8:**
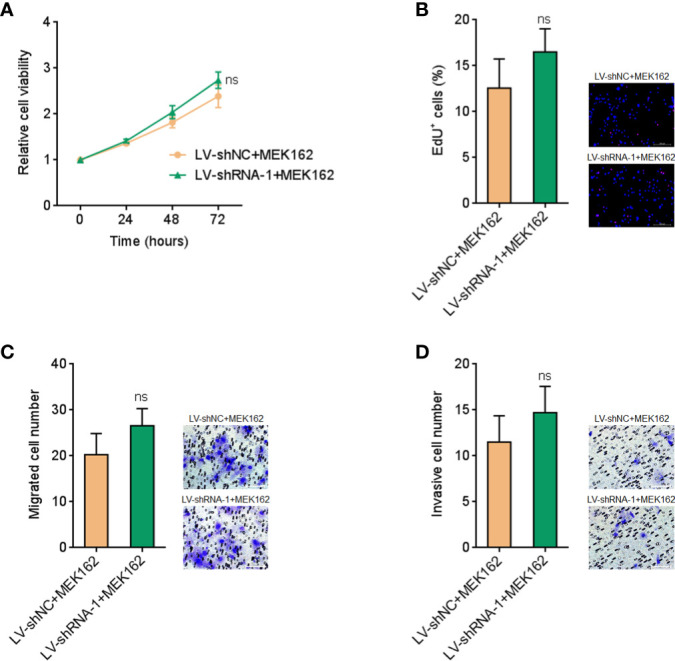
MEK1/2 inhibitor MEK162 abolished the oncogenic roles of FUT8-AS1 silencing in melanoma. **(A)** CHL-1 cells silencing FUT8-AS1 or control were treated with 1 µM MEK162. Then, cell viabilities of treated cells were determined by the Glo cell viability assay. **(B)** CHL-1 cells silencing FUT8-AS1 or control were treated with 1 µM MEK162. Then, cell proliferation of treated cells was determined by the EdU incorporation assay. The red color indicates EdU-positive nuclei. Scale bars, 200 µm. **(C)** CHL-1 cells silencing FUT8-AS1 or control were treated with 1 µM MEK162. Then, cell migration of treated cells was determined by the transwell migration assay. Scale bars, 100 µm. **(D)** CHL-1 cells silencing FUT8-AS1 or control were treated with 1 µM MEK162. Then, cell invasion of treated cells was determined by the transwell invasion assay. Scale bars, 100 µm. Data are presented as mean ± SD. ns, not significant, by two-tailed unpaired t test.

To further elucidate whether the roles of FUT8-AS1 in melanoma were dependent on the regulation of MAPK signaling, we treated FUT8-AS1 silenced and control CHL-1 cells with MEK1/2 inhibitor MEK162. Glo cell viability assays indicated that MEK162 treatment abolished the increased cell viabilities caused by FUT8-AS1 silencing (**Figure 8A**, compared with **Figure 3C**). EdU incorporation assays indicated that MEK162 treatment abolished the accelerated cell proliferation rate caused by FUT8-AS1 silencing (**Figure 8B**, compared with **Figure 3E**). Transwell migration assays indicated that MEK162 treatment abolished the increased migrated cell number caused by FUT8-AS1 silencing (**Figure 8C**, compared with **Figure 3F**). Transwell invasion assays indicated that MEK162 treatment abolished the increased invasive cell number caused by FUT8-AS1 silencing (**Figure 8D**, compared with **Figure 3G**). Thus, these findings suggested that the roles of FUT8-AS1 in melanoma were dependent on the regulation of miR-145-5p/NRAS/MAPK signaling.

## Discussion

Great progressions in molecule targeted therapy and immunotherapy have highlighted the therapeutic strategies based on molecular aberrations in melanoma (43). Current targeted therapies mainly focus on aberrant proteins. Recently, increasing evidences have revealed that except for proteins, ncRNAs also demonstrate important roles in cancers and would be developed as therapeutic targets (4, 44, 45). In this study, *via* analyzing public TCGA dataset, we identified a novel melanoma correlated lncRNA FUT8-AS1. *FUT8-AS1* is locating at chromosome 14q23.3 and has only one exon. The knowledge of FUT8-AS1 is lacking with only one report showing that FUT8-AS1 is correlated with outcome of a subpopulation of glioblastoma multiforme patients (46). In this study, we further investigated the expression, clinical significance, roles, and mechanism of action of FUT8-AS1 in melanoma.

FUT8-AS1 is reduced in melanoma tissues compared with benign nevi. Reduced expression of FUT8-AS1 is correlated with thickness, ulceration, metastasis, and inferior overall survival of melanomas. Gain and loss-of-function assays demonstrated that FUT8-AS1 suppresses melanoma cell proliferation, migration, and invasion *in vitro*. Furthermore, FUT8-AS1 represses melanoma growth and metastasis *in vivo*. Thus, FUT8-AS1 is identified as a tumor suppressive lncRNA in melanoma. FUT8-AS1 would also be a potential prognostic biomarker for melanoma. Enhancing FUT8-AS1 and/or its effects in downstream signaling would be potential therapeutic strategies for melanoma.

In addition, we identified a relative novel molecular mechanism of FUT8-AS1, which is the promotion of miR-145-5p biogenesis *via* competitively binding NF90. Previous reports about the effects of lncRNAs on miRNAs are mainly the competitive binding of miRNAs by lncRNAs, which further relieve the repressive roles of miRNAs on their mRNA targets (47). lncRNA-ATB is a classic example, which competitively binds miR-200s and upregulates miR-200s targets ZEB1 and ZEB2 in hepatocellular carcinoma (48). In our previous report, we have demonstrated that lncRNA MHENCR competitively binds miR-425 and miR-489, and therefore upregulates their targets IGF1 and SPIN1 in melanoma (39). In this study, we found that FUT8-AS1 does not bind miR-145-5p, but promotes miR-145-5p biogenesis.

Combining bioinformatic prediction and experimental verification, we found that FUT8-AS1 directly binds NF90. NF90 is a well-characterized dual strand RNA binding protein. Intriguingly, our previously identified tumor suppressive miR-145-5p was recently reported to be downstream target of NF90 by Zhuang et al. (42). NF90 directly binds pri-miR-145 and represses the biogenesis of mature miR-145-5p (42). In this study, we found that *via* competitively binding NF90, FUT8-AS1 represses the binding of NF90 to pri-miR-145. Therefore, FUT8-AS1 represses the effects of NF90 on miR-145-5p biogenesis. Lastly, FUT8-AS1 downregulates pri-miR-145 and upregulates mature miR-145-5p. The significant positive correlation between FUT8-AS1 and miR-145-5p expression in melanoma tissues supports the positive modulation of miR-145-5p by FUT8-AS1 in human melanoma. Furthermore, functional rescue assays showed that miR-145-5p inhibitors reverse the tumor suppressive roles of FUT8-AS1 in melanoma, supporting miR-145-5p as an important downstream target of FUT8-AS1. Several reports, including our own, have identified NRAS as a critical downstream target of miR-145-5p (29, 49, 50). *Via* targeting NRAS, miR-145-5p further represses MAPK signaling. MAPK signaling has been frequently shown to be aberrantly activated in melanoma (3). Therefore, in this study we further investigated the effects of FUT8-AS1 on NRAS/MAPK signaling *via* regulating miR-145-5p. In line with expectation, our results showed that FUT8-AS1 represses the expression of NRAS and further represses MAPK signaling in melanoma. MAPK signaling inhibitor MEK162 abolished the oncogenic roles of FUT8-AS1 silencing in melanoma, which further supports that miR-145-5p/NRAS/MAPK signaling is the critical mediator of the roles of FUT8-AS1 in melanoma. Therefore, we identified relative novel mechanism of action of lncRNA and also the detailed downstream molecular signaling cascade of FUT8-AS1 in melanoma.

In summary, our findings suggested FUT8-AS1 as an important tumor suppressive lncRNA in melanoma, which represses melanoma cell proliferation, migration, and invasion *in vitro*, and melanoma tumor growth and metastasis *in vivo*. FUT8-AS1 exerts its tumor suppressive roles *via* binding NF90, relieving the repressing roles of NF90 on miR-145-5p biogenesis, upregulating miR-145-5p, repressing NRAS, and lastly repressing MAPK signaling in melanoma. Furthermore, our data and TCGA data both suggested that FUT8-AS1 is correlated with inferior outcome of melanoma patients. Overall, these data provide novel molecular driver of melanoma progression and potential target for melanoma outcome prediction and therapy.

## Data Availability Statement

The raw data supporting the conclusions of this article will be made available by the authors, without undue reservation.

## Ethics Statement

The studies involving human participants were reviewed and approved by the Review Board of the 969^th^ Hospital of PLA. The patients/participants provided their written informed consent to participate in this study. The animal study was reviewed and approved by the Review Board of the 969^th^ Hospital of PLA.

## Author Contributions

Study concept and design: X-JC, SL, LY. Acquisition of data: SL, D-MH, D-ZH, W-JS, X-CZ. Analysis and interpretation of data: X-JC, SL, D-MH, J-QL, LY. Drafting and editing of the manuscript: X-JC, SL. All authors contributed to the article and approved the submitted version.

## Funding

This work was supported by the National Nature Science Foundation of China (81971854).

## Conflict of Interest

The authors declare that the research was conducted in the absence of any commercial or financial relationships that could be construed as a potential conflict of interest.

## Supplementary Material

Supplementary Figure 1 FUT8-AS1 is a noncoding RNA. **(A)** The coding potential of FUT8-AS1 was calculated by the Coding Potential Calculator (CPC). GAPDH and HOTAIR were used as coding and noncoding RNA controls, respectively. **(B)** The coding potential of FUT8-AS1 was calculated by the Coding Potential Assessment Tool (CPAT). GAPDH and HOTAIR were used as coding and noncoding RNA controls, respectively.

Supplementary Figure 2 FUT8-AS1 has a conserved NF90 binding sequence. The structure of FUT8-AS1 was predicted by the mfold web server (http://unafold.rna.albany.edu/?q=mfold). Red line indicates the conserved NF90 binding sequence (5′-CUGUU-3′, 452-456nt of FUT8-AS1).
