# Supplementary figures and images for: FUT8-AS1 Inhibits the Malignancy of Melanoma Through Promoting miR-145-5p Biogenesis and Suppressing NRAS/MAPK Signaling
Source: Front Oncol. 2021 May 19;10:586085. doi: 10.3389/fonc.2020.586085 (PMC8170315; doi:10.3389/fonc.2020.586085)

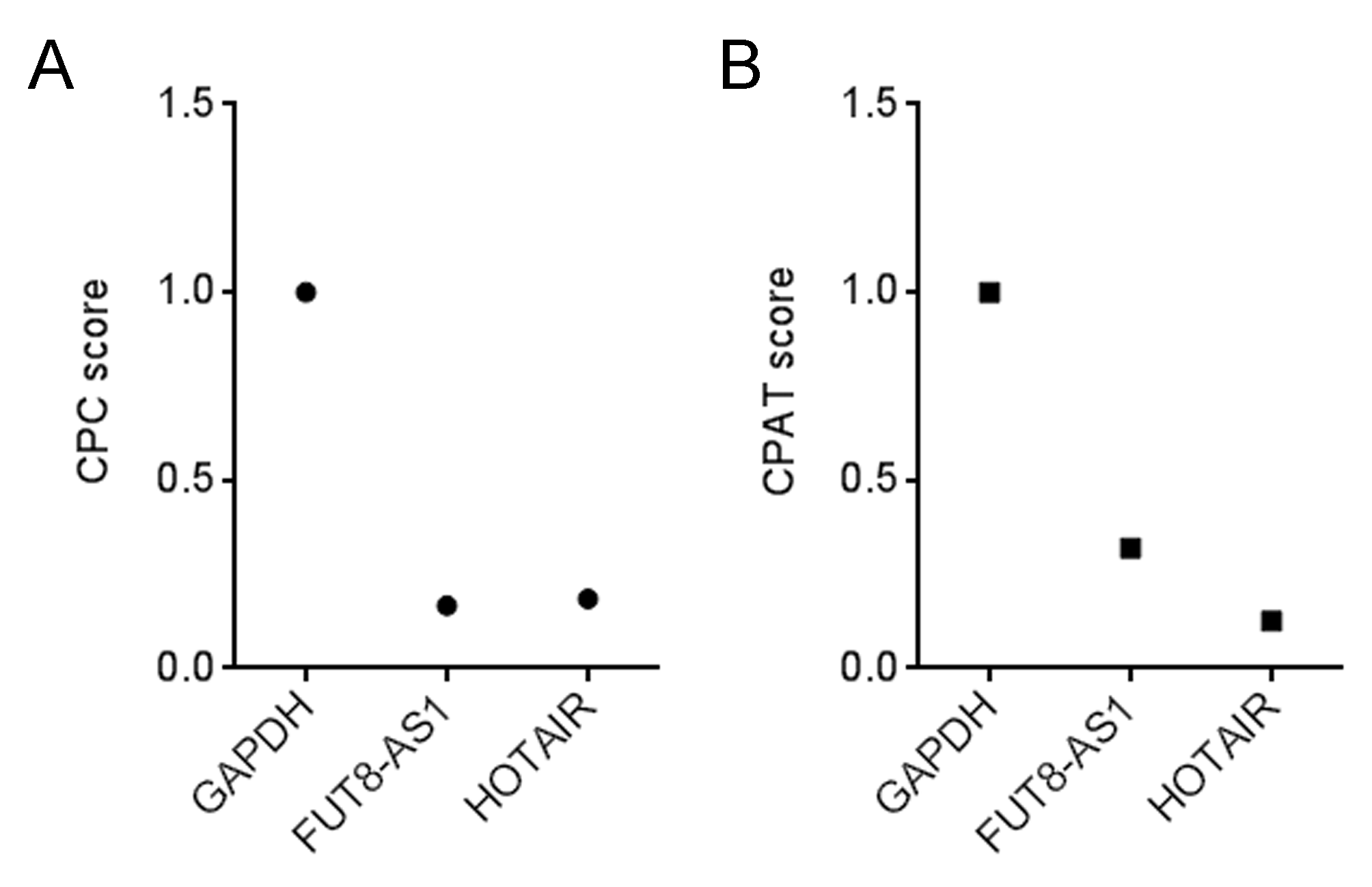

Supplement: Supplementary Figure 1 — FUT8-AS1 is a noncoding RNA. (A) The coding potential of FUT8-AS1 was calculated by the Coding Potential Calculator (CPC). GAPDH and HOTAIR were used as coding and noncoding RNA controls, respectively. (B) The coding potential of FUT8-AS1 was calculated by the Coding Potential Assessment Tool (CPAT). GAPDH and HOTAIR were used as coding and noncoding RNA controls, respectively. [file Image_1.tif]

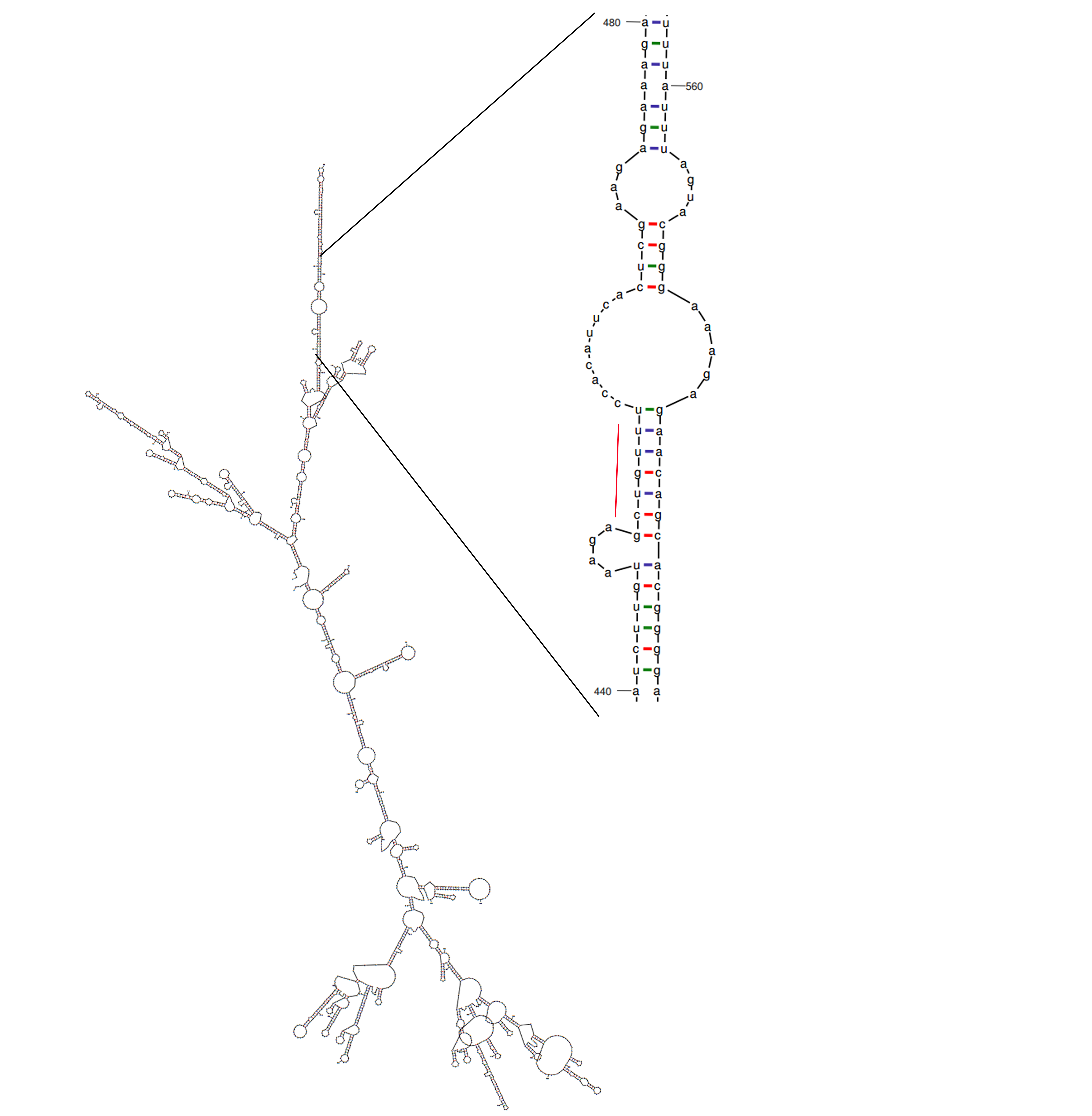

Supplement: Supplementary Figure 2 — FUT8-AS1 has a conserved NF90 binding sequence. The structure of FUT8-AS1 was predicted by the mfold web server (http://unafold.rna.albany.edu/?q=mfold). Red line indicates the conserved NF90 binding sequence (5′-CUGUU-3′, 452-456nt of FUT8-AS1). [file Image_2.tif]

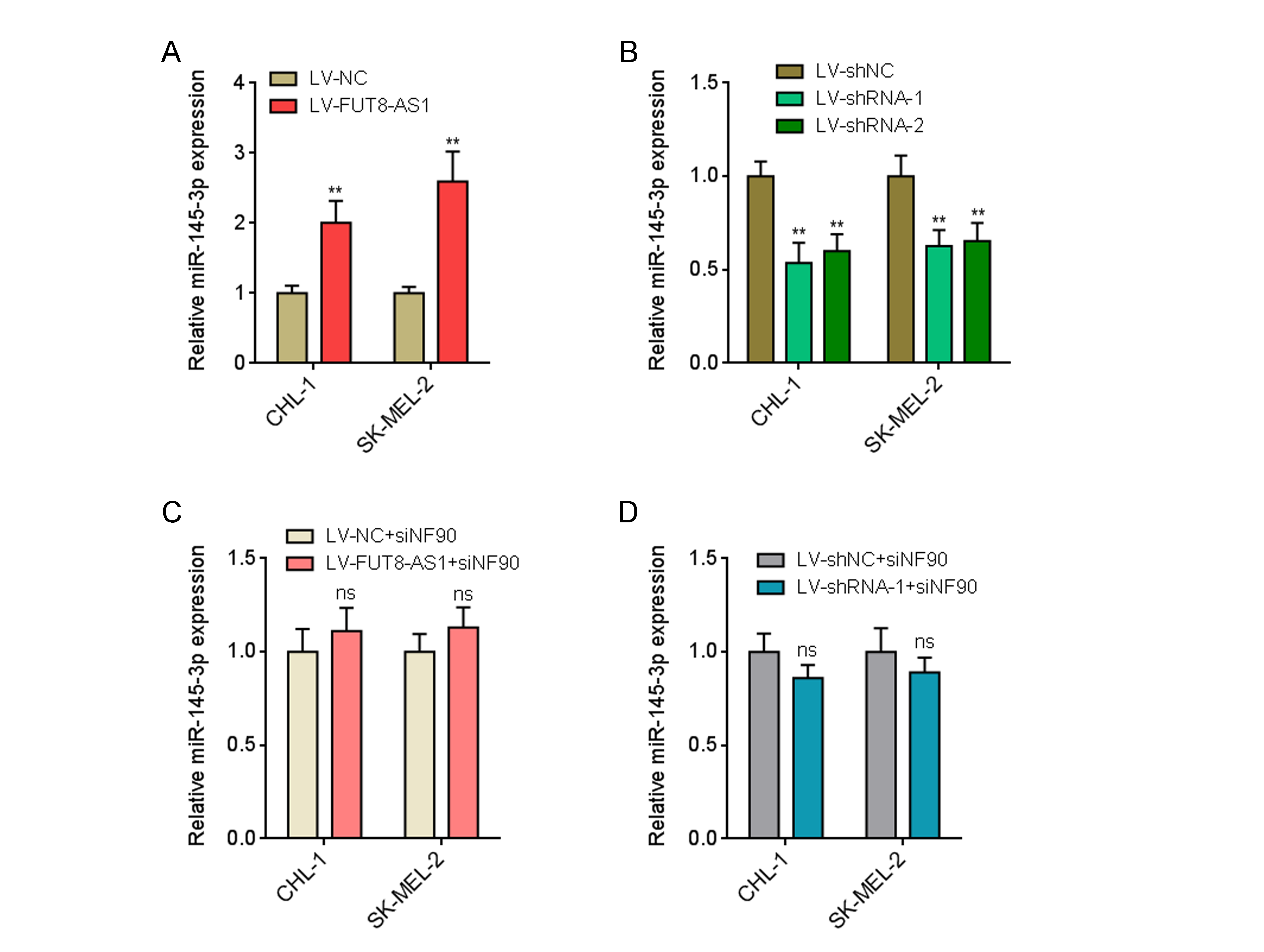

Supplement: Supplementary file 3 [file Image_3.tif]
